# Long-term memory: scaling of information to brain size

**DOI:** 10.3389/fnhum.2014.00397

**Published:** 2014-06-03

**Authors:** Donald R. Forsdyke

**Affiliations:** Department of Biomedical and Molecular Sciences, Queen's UniversityKingston, ON, Canada

**Keywords:** brain scans, computer metaphor, connectionist paradigm, head size, hydrocephaly, information capacity, plasticity limits, ventricle size

## Introduction

“About the truth and extent of these facts none but men possessing a special knowledge of physiology and natural history have any right to an opinion; but the superstructure based on those facts enters the realm of pure reason, and may be discussed apart from all doubt as the fundamental facts” (Jenkin, 1867).

With little modification, these remarks on Darwin's evolution theory by a professor of engineering with little knowledge of biology, will serve to justify the present foray of one not possessing a special knowledge of neurophysiology into the domain of memory.

## Size does not scale with information content

Mother Hubbard spoke authoritatively when she declared the cupboard bare. She knew the size and form of bones likely to satisfy her dog. She also knew the size of her cupboard. Furthermore, she knew that bones were stable, uncompressible and, most importantly, likely to be needed at short notice by her canine friend. We cannot speak on human long-term memory with equal authority. It often appears stable and can be called upon at short notice, but beyond these facts we cannot now go. We can, however, speak about the size of the metaphorical “cupboard” where, conventional wisdom holds, long-term memory must lie (Draaisma, 2000). And if the cupboard proves to be bare, we have to admit a problem (Forsdyke, 2009).

The cranial “cupboard” might be expected to be larger than normal in individuals (savants) with very large long-term memories. Although some savants are deficient in other respects (savant syndrome), the discovery of just one savant with exceptionally high long-term memory and with normal cranial capacity, but without serious deficiency, would refute this prediction. It so happens that, although one much celebrated savant had a large head, most savants have not, and some show no serious deficiency; on the contrary, they show some exceptional abilities, including exceptionally high long-term memory (Treffert, 2010). Conversely, when the cranium is much smaller than normal (microcephaly), long-term memory might be decreased. Unfortunately, microcephalics tend to be tested more for intelligence (usually impaired), than for their ability to recall information. However, on the assumption that intelligence and long-term memory are to some extent related, we can note that a few microcephalics have normal intelligence (Forsdyke, 2009). Thus, with both savants and microcephalics, the evidence, albeit weak, suggests a *disconnect* between the volume of neural tissue held within the cranium and the quantity of information which that neural tissue is, in some way, held to store. *Size does not matter*. Studies of hydrocephalics are casting new light on this scaling paradox.

## Adult hydrocephalics with 5% brain tissue by volume

With savants and microcephalics, the volume of neural tissue is determined by cranial capacity. However, with hydrocephalics the volume is largely determined by the size of the fluid-filled ventricles. A therapeutic shunt in early life can lower a cerebrospinal fluid pressure that otherwise would relentlessly compress neural tissue against the cranial surface. In the 1970s innovations in non-invasive brain-scanning technology facilitated the reexamination in adult life of treated hydrocephalics. The journal *Science*, under the title “Is your brain really necessary?” (Lewin, 1980), described a series of 600 cases with residual ventricular enlargement that had been studied in Britain by paediatrician John Lorber (1915–1996). Again, while long-term memories were not directly assessed, intelligence quotients (IQs) were.

Amazingly, in 60 of Lorber's cases, ventricular fluid still occupied 95% of cranial capacity. Yet half of this group had IQs above average. Among these was a student with an IQ of 126 who had a first class honors degree in mathematics and was socially normal. For this case Lorber noted:

“Instead of the normal 4.5 cm thickness of brain tissue between the ventricles and the cortical surface, there was just a thin layer of mantle measuring a millimeter or so. The cranium is filled mainly with cerebrospinal fluid. … I can't say whether the mathematics student has a brain weighing 50 or 150 g, but it's clear that it is nowhere near the normal 1.5 kg.”

**Figure 1:**
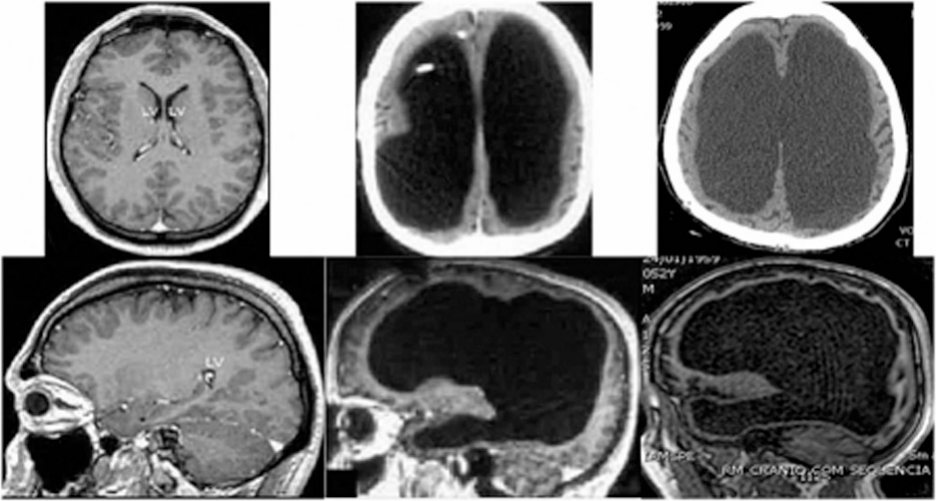
**Brain scans of patients of de Oliviera et al. (2012)**. Normal adult appearance (left), with “LV” referring to the small black fluid-filled ventricles. Enlarged ventricles (middle and right). The middle patient is clinically normal, whereas the right patient has had “deep cognitive and motor impairment since childhood.” Reproduced with author's permission from *Frontiers in Human Neuroscience* under Creative Commons Licence.

Lorber's findings met much skepticism. But recently there have been two independent confirmations, suggesting Lorber should not have been so lightly dismissed. Under the title, “Brain of a white-collar worker,” French neurologists (Feuillet et al., 2007) showed “massive ventricular enlargement” in the brain scan of a civil servant who had an IQ in the low normal range and came to them with relatively mild neurological symptoms that responded to treatment. Shortly thereafter, neurosurgeons in Brazil reported a similar case (de Oliviera et al., 2012). Their figure (Figure 1) is striking, since it compares the enlarged ventricles of their symptom-free subject with the equally enlarged ventricles of a subject with “deep cognitive and motor impairment”—the more usual expectation.

## Plasticity or paradox?

Data from savants, microcephalics, and hydrocephalics seem to be telling us that, with respect to long-term memory, there are circumstances where, paradoxically, size does not matter. Lorber suggested that “primitive deep structures that are relatively spared in hydrocephalus” may have allowed his subjects to live normal lives, so that “there must be a tremendous amount of redundancy or spare capacity in the brain.” This implied that normally much of the brain is simply idling, ready to act as a backup should the need arise.

Somewhat more convincing was a “plasticity” explanation advanced by Bateson and Gluckman (2011) when commenting on the French patient. In similar fashion, the Brazilian team invoked the “resilient adaptation of brain networks” associated with “the ability of neuronal tissue to reassume and reorganize its functions” (de Oliviera et al., 2012). These plasticity explanations imply that, in keeping with the sometimes amazing recoveries reported for severe brain injuries, an otherwise-occupied part of the brain can change to compensate for a defective part. Under the prevailing neural network “connectionist” paradigm (Draaisma, 2000; Forsdyke, 2009), for hydrocephalics with only 5% of neural tissue remaining, this would seem to require the establishment early in life of a critical network, which would have to retain its connectivity in the face of the subsequent severe progressive distortion associated with ventricular expansion. Unfortunately, there are no post-mortem histological studies on this.

However, there must be rules for redundancy and plasticity. There *must be limits*. It is a matter of elementary logic that, at some stage of brain shrinkage, these explanations must fail (Majorek, 2012). The drastic reduction in brain mass in certain, clinically-normal, hydrocephalic cases, seems to demand unimaginable levels of redundancy and/or plasticity—*superplasticity*. How much brain must be absent before we abandon these explanations and look elsewhere? Perhaps we are looking a gift paradox in the mouth?

The extent of our neurobiological ignorance was recently noted by Eric Kandel (2006): “In the study of memory storage, we are now at the foothills of a great mountain range. … To cross the threshold from where we are to where we want to be, major conceptual shifts must take place.” Regarding the human brain's “massive storage capacity” for object details, Brady et al. (2008) have also challenged “neural models of memory storage and retrieval.” Others are calling for “radical modification of the standard model of memory storage” (Fusi and Abbott, 2007; Firestein, 2012). Given the doubts of these specialists, perhaps it is time for other hypotheses to be admitted to the table of responsible neuroscientific discourse. Should we not be looking further afield—alert for rare gift horses (van Heerden, 1968; Pribram, 1991; Talbot, 1991; Berkovich, 1993)? Metaphors may help.

## From stand-alone to cloud computing

In 1934 librarian Paul Otlet (1868–1944) envisioned a “mechanical collective brain” to which individuals could connect through “electric telescopes” that would seem to equate with today's personal computers (Levie, 2006). And in 1970 at the first international conference on “Man and Computer,” computer engineer John McCarthy (1972) sketched out how the “home informational terminal”—a console—might one day retrieve personal files from, and store personal files to, a central resource—now known as “the cloud.” However, when personal computers appeared in the 1980s they were essentially stand-alone, with their own software and data-storage (memory). It was easy to relate this, metaphorically, to the perception that an individual human brain is a stand-alone entity, with its own software and memory (Draaisma, 2000; Noll, 2003). Indeed, concerning “generalized cognition-space,” Lenneberg (1965) had pointed to doubts raised by logical analogy:

“There is, however, another line of argument that induces many scholars to suspect a close relationship between brain size and intelligence. It is based on purely logical considerations; in fact, the reasoning underlying it is by analogy. The capacities of an electronic computer or desk calculator are directly related to the number of its constituent elements. This engenders the belief that an increase in the number of units in the brain has a similar consequence. However, evidence on this is surprisingly poor. … Although it is entirely possible that the emergence of language and intelligence are historically related to the increase in size of the brain, the case is certainly not yet irrefutably proven.”

The known stand-alone memory storage alternatives have been reviewed elsewhere. For example, although speculation continues, the idea that long-term memory might reside in brain DNA is largely put to rest (Forsdyke, 2009). The question of a role for non-DNA polymers remains. Broadly, this category includes other macromolecules (RNA, protein, lipid, carbohydrate), or unknown subatomic forms perhaps related to “quantum computing” (Sciarrino and Mataloni, 2012). Also in this category is the attractive idea of the brain as a three dimensional holographic storage network (van Heerden, 1968; Pribram, 1991; Draaisma, 2000). But, before seeking such exotic storage modalities, we should ensure that the brain “cupboard” is indeed bare of forms more in keeping with current paradigms. We need a better inventory of brain-specific macromolecules to exclude a polymeric form that, DNA-like, might store information digitally (Tsien, 2013). Furthermore, although brain information might be stored in some subatomic form, at some point that form would need to interface with more conventional macromolecular species (e.g., proteins). Specific adaptations for this role should distinguish them from other macromolecules.

For all these storage alternatives, the thinking is conventional in that long-term memory is held to be *within* the brain, and the hydrocephalic cases remain hard to explain. Yet currently most of us, including the present author, would prudently bet on one or more of the stand-alone forms. The unconventional alternatives are that the repository is external to the nervous system, either elsewhere within the body, or extra-corporeal. The former is unlikely since the functions of other body organs are well understood. Remarkably, the latter has been on the table since at least the time of Avicenna and hypothetical mechanisms have been advanced (Talbot, 1991; Berkovich, 1993; Forsdyke, 2009; Doerfler, 2010). Its modern metaphor is “cloud computing.”

Even though the internet emerged in the 1990s (Berners-Lee, 2010), it took two decades for cloud computing to become established (Furht, 2010). Imaginative attempts to relate this to the workings of individual brains (Talbot, 1991; Berkovich, 1993), still fall far short on evidence (Forsdyke, 2009). However, the rare hydrocephalic cases described here suggest we should exercise caution when tempted to cast aside the astonishing idea of personal information—long-term memory—being remotely stored. After all, Nature is not obliged to conform to our preconceptions. And, as Sherlock Holmes once said, “when you have eliminated the impossible, whatever remains, however improbable, must be the truth.”

The importance of this extends far beyond neuroscience and the clinic. When speaking of extracorporeal memory storage we enter the domain of “mind” or “spirit,” with corresponding metaphysical implications (Crick, 1995; Draaisma, 2000; Forsdyke, 2009). We begin to “secularize the soul” (Hacking, 1995). Thus, there may be vestiges of truth amongst the dross that we poor creatures, imprisoned within the second decade of the twenty-first century, can comprehend no better than those imprisoned in the later decades of the nineteenth century would have comprehended Gregor Mendel, had they known of him (Cock and Forsdyke, 2008). And that which is now deemed metaphorical may not always remain so. Draaisma (2000) notes that metaphors can die and become literal. There are those who urge us to lift our eyes to new horizons (Talbot, 1991; Berkovich, 1993). While they may lack a formal training in neuroscience, we should listen carefully.

## Summary

The material bases of information—paper, computer discs—usually scale with information quantity. Large quantities of information usually require large material bases. Conventional wisdom has it that human long-term memory locates within brain tissue, and so might be expected to scale with brain size which, in turn, depends on cranial capacity. Large memories, as in savants, should always require large heads. Small heads should always scale with small memories. While it was previously concluded that neither of these predictions was invariably true, the evidence was weak. Brain size also depends on ventricle size, which can remain large in some survivors of childhood hydrocephaly, occupying 95% of cranial volume. Yet some of these have normal or advanced intelligence, indicating little impairment of long-term memory. This paradox challenges the scaling hypothesis. Perhaps we should be looking further afield?

### Conflict of interest statement

The author declares that the research was conducted in the absence of any commercial or financial relationships that could be construed as a potential conflict of interest.
